# Supplementary material for: Micro Scalable Graphene Oxide Productions Using Controlled Parameters in Bench Reactor
Source: Nanomaterials (Basel). 2021 Jul 31;11(8):1975. doi: 10.3390/nano11081975 (PMC8398880; doi:10.3390/nano11081975)

Micro Scalable Graphene Oxide Production Using Controlled Parameters in Bench Reactors

Supplementary Materials

S1 - EXPERIMENTAL DETAILS

Table S1: mG/mROxi ratios.

Sample	Graphite mass (g)	H_2SO_4/H_3PO_4 (mL)	$KMnO_4$ mass (g)
1	3.0	360/40	18.0
2	4.5	360/40	18.0
3	6.0	360/40	18.0

The experimental parameters were based on the heat exchange between the reactor and the reaction medium, the change in viscosity that occurs during synthesis and the need to make a fast dispersion of the product after the addition to the system. The method used in this choice of standardization was “trial and error”, where the synthesis was repeated several times until finding the best results. The GO samples were transferred individually to 3.0 L beakers and left for 24 hours for decantation. After this period, the supernatant was removed and 1.0 L of 1:10 HCl solution was added, followed by another 24 h decantation period and the supernatant was again removed and discarded. The precipitate was fractionated into 50 mL Falcon tubes, washed exhaustively with deionized and centrifuged in a DT 4500 Daiki centrifuge. In each washing step, the supernatant was removed, deionized water was added to the tube until its total volume and the content was homogenized and centrifuged for 20 minutes at 4500 pm. This procedure was repeated until the pH of the medium reached values between 5 and 6. After washing, the GO samples were frozen at -23 ºC and lyophilized for 96 h. After the lyophilization, they were transferred to glass bottles, sealed with parafilm and kept in the desiccator. For the preparation of GO, 1 mg/mL dispersions were prepared. The graphite oxide samples were exfoliated in an ultrasound bath (Elmasonic P30H) for 60 minutes, 320 W and 37 Hz. For instance, figure S1(a-c) shows GO 3g sample dispersions in three different concentrations. Based on previous experiments, the temperatures and rotation parameters were determined as presented on Table S2.

Table S2: Parameters adopted for the bench reactor during the GO production, where T is temperature and T_{max} is maximum temperature.

**Step**	**Description**	**Rotation (RPM)**	**Adjusted T(°C) (± 0.1)**	**T_{max} reached (°C) (± 0.1)**
1	Initial	100	5.0	5.0
2	Mixture of acids	250	5.0	5.0
3	Graphite addition	300	0.0	4.0
4	$KMNO_4$ addition	450	0.0	3.0
5	Oxidation pause	150	50.0	50.5
6	Dilution in deionized water	450	0.0	50.0
7	Peroxide addition	450	10.0	15.0

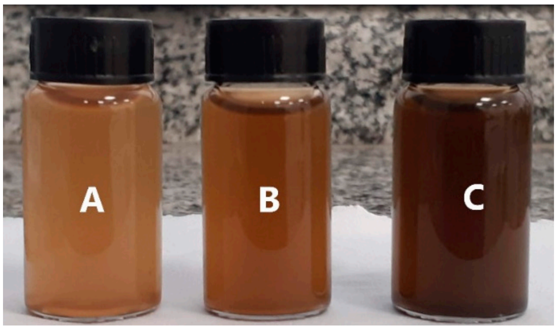

Figure S1: GO 3.0g dispersions in different concentrations: (**a**) 1 mg/mL,(**b**) 3mg/mL, and (**c**) 10mg/mL.

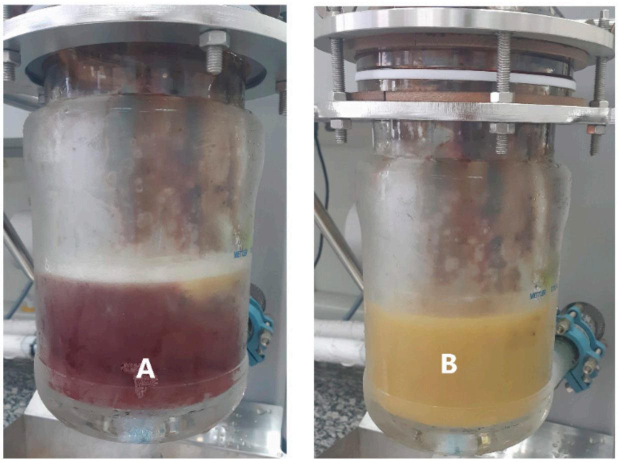

Figure S2: Addition stages of hydrogen peroxide: start point (a); final point (b) after changing color.

A representative SEM image of GO sample (GO 9.0 g) can be seen in Figure S3-a, where the morphology of the GO powder grain constituted by highly agglomerated flakes is observed. The representative Raman spectra for graphite and GO 9.0g samples are shown in Figure S3-b. The main Raman characteristic bands for graphitic materials are shown (D, G and G' bands). The G band that appears at ~1582 cm^{-1} is characteristic of the tangential vibrational modes of graphite, the D band (~1350cm^{-1}) is associated with vibrational modes resulting from structural disorder from defects, functionalizations and sp^3 carbons. The G' band (~2700cm^{-1}) is associated with the second order vibrational mode of the D band, and it can be used to differentiate graphene samples with different amounts of graphitic layers. For this produced sample, an increase in the ID/IG ratio from 0.43 to 0.75 in relation to the graphite was observed, resulting from the oxidative process.

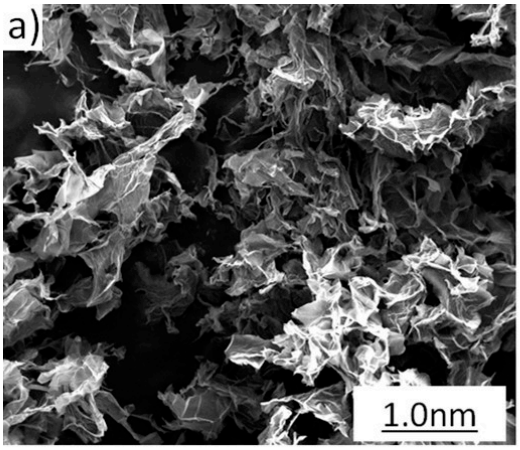

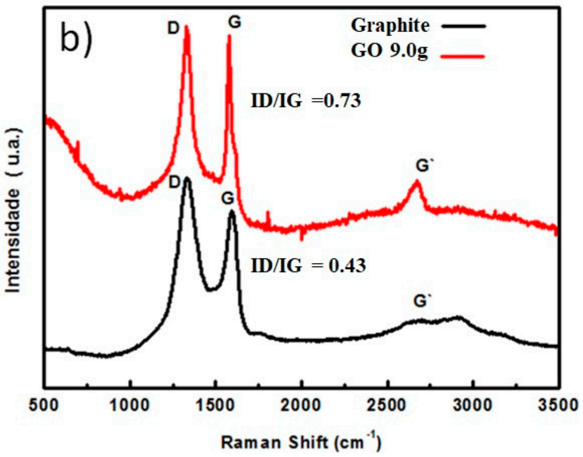

Figure S3: (a) SEM image of GO 9.0 g multilayer, (b) Raman spectra for graphite and GO 9.0g multilayer samples.

Figure S4a-b shows the AFM profile of the GO 9.0g multilayer sample with micrometric size. In this case, the nanosheets do not presented well-defined edges, but different regions of contrasts with lateral sizes of up to ~0.5µm and graphitic layers between 4-8.

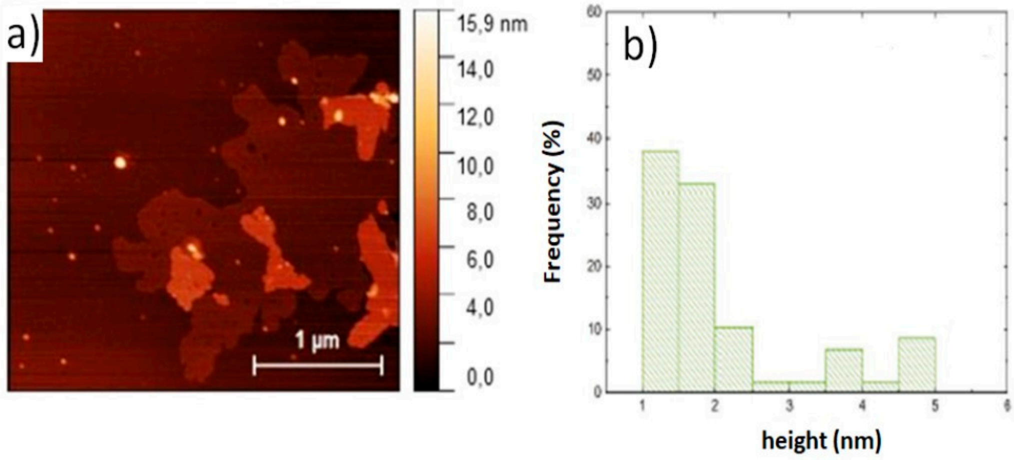

Figure S4: AFM image of GO 9.0g multilayer sample (a), height profile (b).

In figure S5 four distinct regions of mass loss can be observed, the first in the region between 25-100ºC which is related to adsorbed solvent loss on the surface of the sample. There are two events between 150-300ºC related to the loss of mass of different oxygenated functional groups. Finally, in the region between 400-600ºC, there is a loss of mass relative to the graphitized part of the sample.

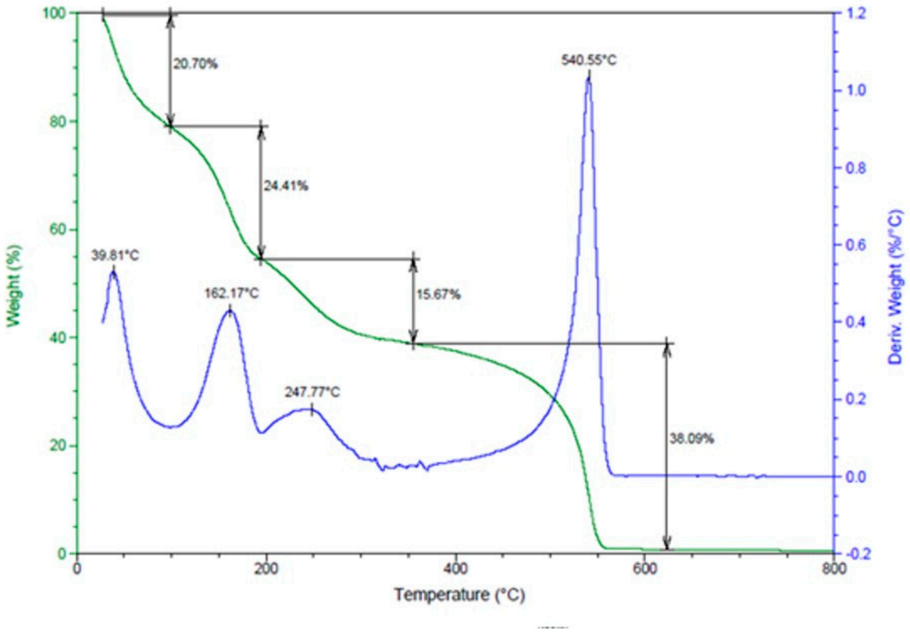

Figure S5: TG/DTG curves of GO 9g multilayer sample.

Figure S6: FTIR bands of GO samples and its respective wavelength.

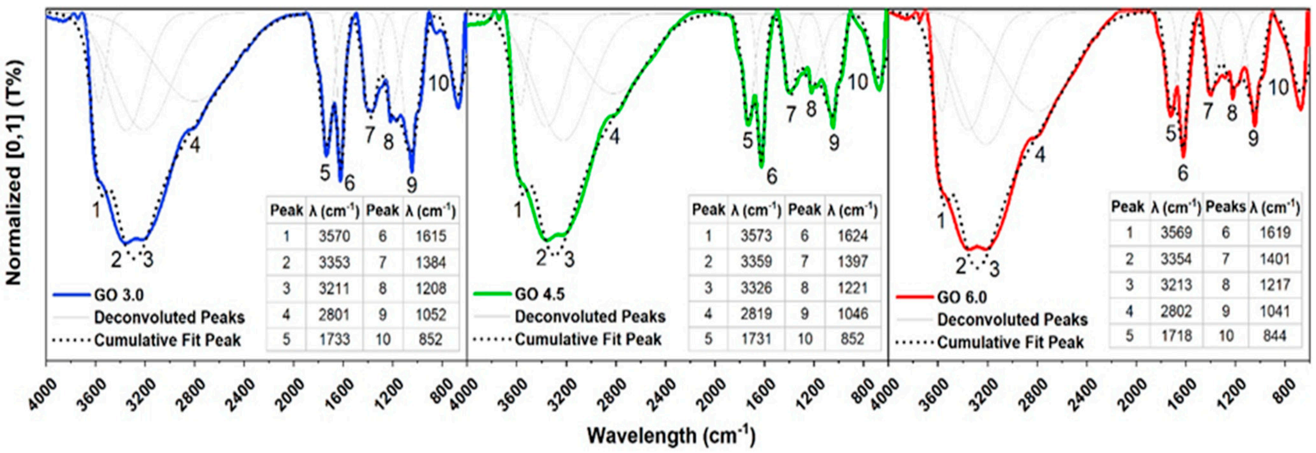

Supplement: Supplementary file 1 [file nanomaterials-11-01975-s001.zip › nanomaterials-1297265-supplementary.pdf]
